# Case report: Massive hepatocellular carcinoma with complete response to the non-surgical systematic treatment strategy

**DOI:** 10.3389/fonc.2024.1291131

**Published:** 2024-05-10

**Authors:** Yun Li, Yanzhen Lai, Xuqiang Luo, Jian Wu, Kunpeng Wu, Haiqing Ma

**Affiliations:** ^1^ Department of Oncology, Heyuan Hospital of Guangdong Provincial People’s Hospital, Heyuan People’s Hospital, Heyuan, Guangdong, China; ^2^ Heyuan Key Laboratory of Molecular Diagnosis & Disease Prevention and Treatment, Doctors Station of Guangdong Province, Heyuan People's Hospital, Heyuan, Guangdong, China; ^3^ Medical Research Center, Medical Research Institute, Guangdong Provincial People’s Hospital (Guangdong Academy of Medical Sciences), Southern Medical University, Guangzhou, Guangdong, China

**Keywords:** hepatocellular cancer, radiotherapy, complete response, anlotinib, sindilizumab

## Abstract

**Background:**

The five-year recurrence rate of hepatocellular carcinoma (HCC) remains as high as 70%. A complete clinical response has not been observed without surgical resection. Here, we report a rare case of clinical complete response and long-term survival in a patient with massive HCC receiving treatment with immunotherapy, anti-angiogenic therapy, and radiotherapy.

**Case description:**

A 38-year-old woman presented to our hospital for abdominal pain that persisted for 3 months. She was diagnosed as Barcelona Clinic Liver Cancer(BCLC) stage A, with a Cancer of the Liver Italian Program (CLIP) score of 3, American Joint Committee on Cancer (AJCC) Tumor-Node-Metastasis (TNM) staging systems stage IB. She refused surgical resection and trans-arterial chemoembolization and accepted a non-invasive systematic treatment strategy involving immunotherapy, anti-angiogenic therapy, and radiotherapy. Her tumor burden decreased, and she experienced partial response before radiotherapy. Following radiotherapy, she experienced a complete clinical response and has been alive for more than 36 months after her initial presentation. She is currently alive.

**Conclusion:**

A non-invasive systematic treatment strategy is a potential radical treatment option for patients with massive HCC.

## 1 Introduction

Hepatocellular carcinoma (HCC) is a common cancer worldwide and currently the third leading cause of cancer-related deaths (1, 2). A multidisciplinary approach involving surgical resection, radiotherapy, trans-arterial chemoembolization, or liver transplantation has led to good response in patients with HCC. However, the five-year recurrence rate remains as high as 70% (3), and complete clinical response (CCR) has not been observed without surgical resection. Here, we present a case of long-term survival in a patient with massive HCC treated with immunotherapy, anti-angiogenic therapy, and radiotherapy.

## 2 Case description

**Figure 1:**
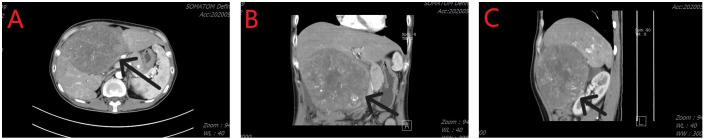
Computed tomography imaging revealed a hepatic tumor mass. **(A)** Axial image; **(B)** Coronal image; **(C)** Sagittal image.

On May 6, 2020, a 38-year-old woman presented to our hospital due to abdominal pain that persisted for 3 months. She has had chronic hepatitis B for over 30 years and had not received treatment for the condition. She had no other underlying disease. On assessment of her performance index according to the Eastern Cooperative Oncology Group scoring system, she had a score of 1. Initial laboratory investigations showed the total bilirubin level was 1.93 mg/dL, albumin was 3.9 g/dL, and prothrombin time international normalized ratio was 1.13. As shown in **Figure 1**, a computed tomography (CT) scan performed at the time of her presentation showed a solitary hepatic mass measuring 160 mm × 110 mm, with no other nodules. There was no tumoral thrombosis in the other hepatic lobe, supra-hepatic inferior vena cava, or right atrium. The baseline alpha-fetoprotein (AFP) level was > 3000 ng/mL, and the details of the tumor staging include Barcelona Clinic Liver Cancer stage A, Cancer of the Liver Italian Program score of 3, and American Joint Committee on Cancer Tumor-Node-Metastasis stage IB.

**Figure 2:**
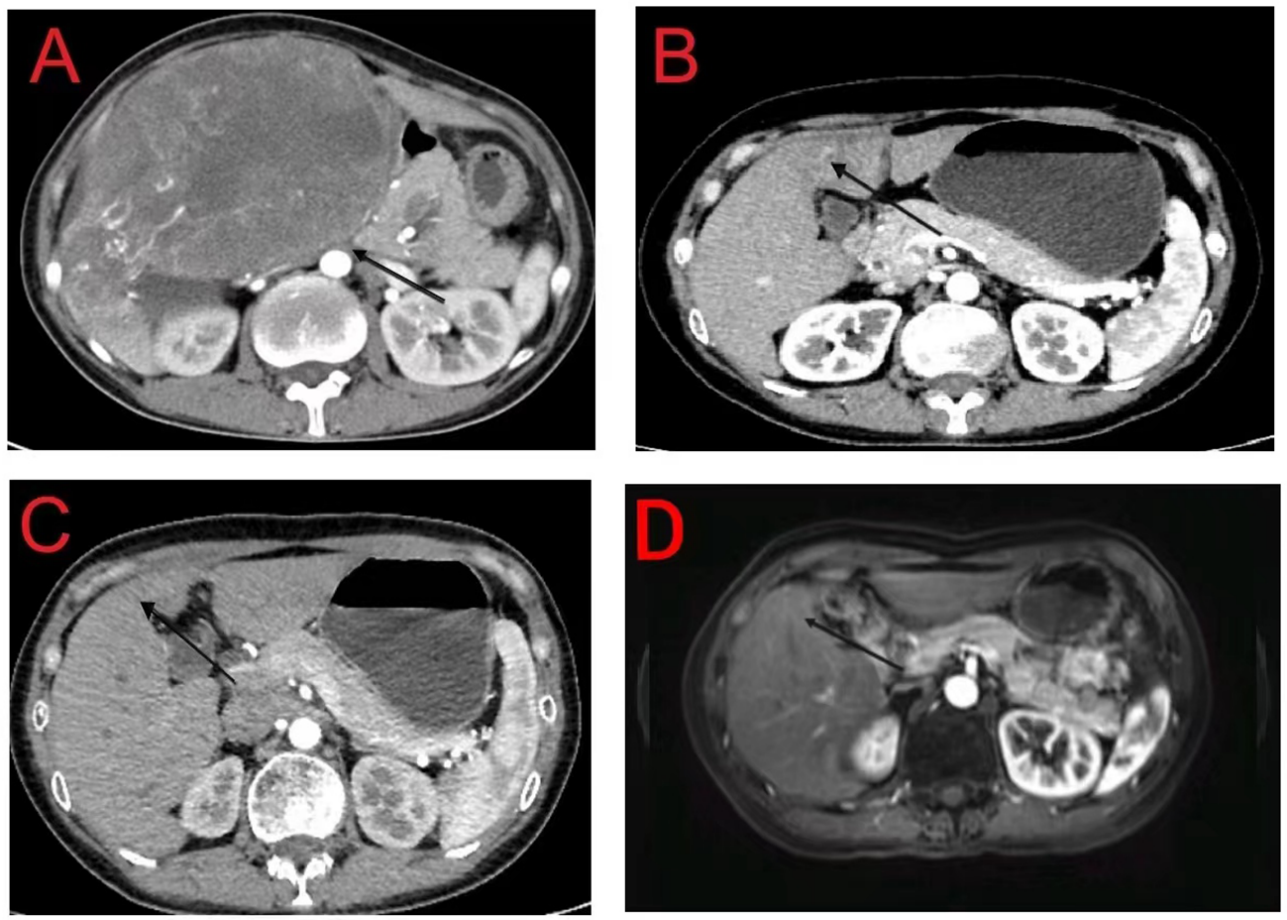
**(A)** Initial computed tomography (CT) scan of the abdomen showing a 160 mm × 110 mm hepatic mass with no daughter nodules in the other hepatic lobe or tumoral thrombosis in the inferior vena cava and right atrium; **(B)** Tumor mass is reduced to 37 mm × 31 mm following neoadjuvant treatment; **(C, D)** CT and magnetic resonance imaging reveal post-treatment changes in the junctional area of the left and right lobes of the liver with no recurrent lesions.

**Figure 3:**
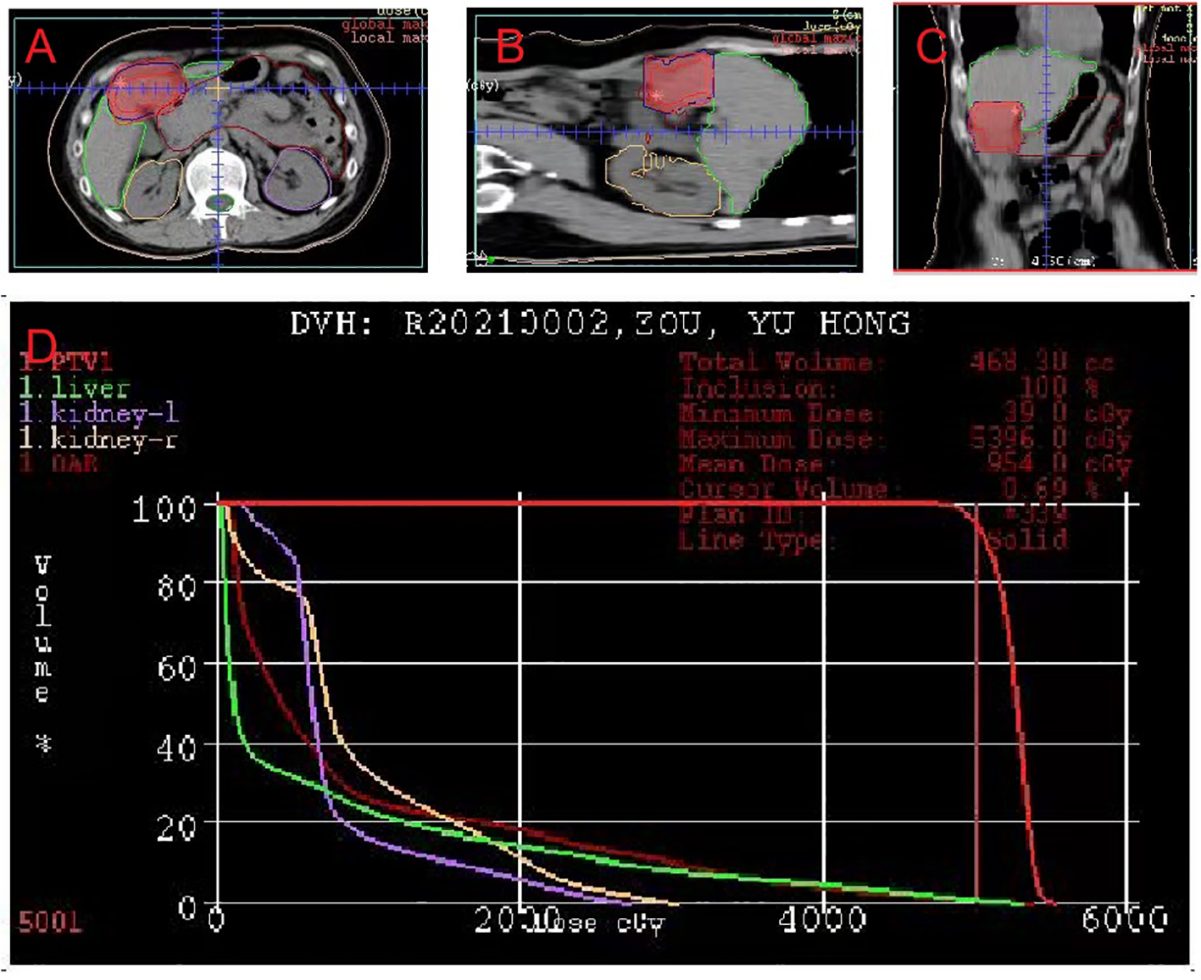
Dose-planning of radiotherapy. Planned target volume (GTV: red, PTV1: blue) covered by the 95% isodose can be seen in **(A)** axial, **(B)** coronal, and **(C)** sagittal planes on pretreatment planning computed tomography. **(D)** Dose-volume histogram shows that 95% of the PTV volumes irradiated with more than 5000 cGy.

**Figure 4:**
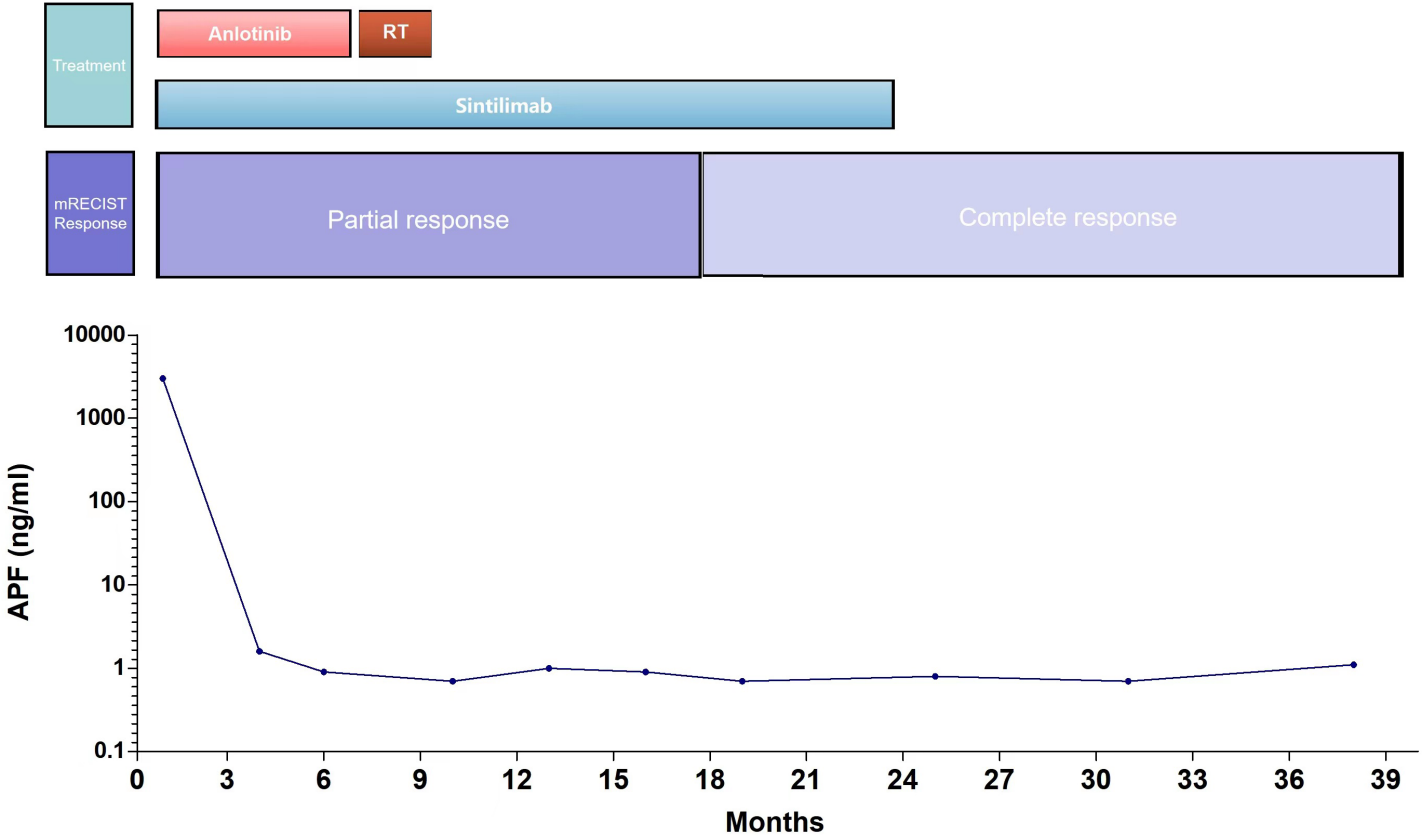
The clinical course of the patient.

After the diagnosis, she declined surgical resection and trans-arterial chemoembolization. However, she received seven courses of sindilizumab 200 mg plus anlotinib 12 mg as neoadjuvant therapy prior to radiotherapy. The AFP level decreased to 1.8 ng/mL based on investigations carried out on September 22, 2020. Follow-up CT scan revealed a tumor size of 37 mm × 31 mm (**Figures 2A, B**). Based on the response evaluation criteria in solid tumors, a partial response was observed. On January 20, 2021, she commenced daily radiation therapy (total 5000 cGy in 25 sessions) for the residual hepatic disease (**Figures 3A–D**) with concurrent immunotherapy (sindilizumab 200 mg). On December 20, 2022, CT and magnetic resonance imaging scans revealed post-treatment changes of HCC in the junctional area of the left and right lobes of the liver (**Figures 2C, D**). No significant recurrence was observed, and a complete response was noted. Maintenance therapy of 200 mg sindilizumab was administered until July 2022. The patient was still alive more than 36 months after diagnostics at our hospital and has returned to everyday life and work (**Figure 4**).

## 3 Discussion

There has been no study reporting CCR and long-term survival in a patient with massive HCC treated with immunotherapy and anti-angiogenic therapy prior to radiotherapy. A point to note in the case was that the tumor mass of the patient decreased gradually. Post-treatment, she eventually achieved CCR with an overall survival of 36 months longer than that obtained in other trials. The satisfactory therapeutic effect may be explained by the following.

Effective management of HCC relies on medical and locoregional therapies. Neoadjuvant therapy downstages the tumor to increase the effect of locoregional treatment, which facilitates a decrease in the probability of recurrence through the seeding of micrometastasis after treatment (4, 5). Ho et al. demonstrated the efficacy of nivolumab and cabozantinib in downstaging tumors to enable potential resection in a trial enrolling 15 patients with HCC who did not qualify for surgical resection due to the presence of multifocal disease, high-risk of portal vein invasion, or tumor diameter >10 cm. Following downstaging, 12 patients had a successful surgical resection, with a major pathologic response (defined as ≥ 90% necrosis) noted in half, whereas one patient had a complete pathologic response (6).

Moreover, a small randomized phase II trial validated that perioperative immunotherapy (nivolumab with or without ipilimumab) achieved a notable complete pathological response (29%) without causing a delay in surgical resection (7). Yarchoan et al. reported that cabozantinib combined with nivolumab induced a marked pathologic response and enabled surgical resection in patients who initially did not qualify based on traditional criteria (8). Here, sintilimab (Innovent Biologics, Suzhou, China), a highly selective humanized, monoclonal antibody, was used. Some studies have employed sintilimab plus a bevacizumab biosimilar (IBI305), which has been currently approved in China as first-line for unresectable or advanced HCC (9–11). Anlotinib is a novel multi-targeting tyrosine kinase inhibitor that inhibits vascular endothelial growth factor receptor, platelet-derived growth factor receptor α/β, fibroblast growth factor receptor 1-4, and c-kit (12). Findings from ALTER0802 showed that anlotinib is a valid and safe first- or posterior-line treatment for HCC (13). Moreover, a single-arm phase II study utilizing a combination of sintilimab and anlotinib as first-line therapy in patients with advanced HCC reported an objective response rate of 42.9%, with complete and partial response in 1 and 5 of 14 patients, respectively. Stable disease was noted in 7% of all patients, and the disease control rate was 92.9% (13 of 14 patients). The median duration of response was not reached (95% confidence interval [CI]: 9.0 months–not reached), and all responses were sustained at the time of study completion. The 6-month progression-free survival (PFS) rate was 78.8% (95% CI: 38.1%–94.3%), and the median PFS was not reached (14). The combination of sintilimab and anlotinib is a promising option with manageable toxicity as a first-line or posterior-line treatment of HCC. Studies are ongoing to improve and validate this combination regimen. Overall, previous trials enrolled patients with advanced stages and employed immunotherapy and anti-angiogenic therapy for optimizing survival. In our case, seven courses of a combination of sindilizumab 200 mg and arlotinib 12 mg reduced the dimension of her hepatic mass from 160 mm×110 mm to 37 mm×31 mm after patient’s refusal to undergo surgical resection and trans-arterial chemoembolization. Her serum AFP level also reduced from >3000 ng/ml to 1.8mg/ml.

Radiotherapy and chemoembolization are feasible and preferable loco-regional options, and either of them is recommended for local disease control when surgical treatment is contraindicated (15). In our case, radiotherapy was an indispensable tool for delivering high doses of radiation to the tumor while limiting exposure to the surrounding healthy tissues (16). In a phase II prospective clinical trial, 128 patients with either hepatobiliary cancers or liver metastases with a median tumor volume measuring 10 cm were given high doses of three-dimensional conformal radiation therapy with a maximum of 90 Gy (median, 60.75 Gy) in twice-daily sessions of 1.5 Gy each. Participants also received concurrent hepatic artery infusion of foxuridine. The median survival for control patients with HCC was 8 months versus 15.2 months in the trial. Radiation-induced liver toxicity was observed in 4% of all patients (17, 18). Sapir et al. reported outcomes of a propensity score analysis of 209 patients with one or two tumor nodules who underwent transarterial chemoembolization (n=84) or radiotherapy (n=125). The 2-year local control rate was superior with radiotherapy compared with chemoembolization (91% vs. 23%), with similar survival rates at 2 years (overall survival: 34.9% vs. 54.9%) (19). In some countries’ guidelines, radiotherapy is a prioritized treatment for patients with early, intermediate, and advanced HCC. In other countries, it is reserved as a treatment option only if other standard treatments are not feasible (20).

Our patient was still alive at 36 months after diagnostics, and this demonstrates our non-invasive systematic approach as a potential radical option in massive HCC. As far as we know, there are no published clinical research data on the use of combined immunotherapy and radiotherapy to treat HCC. However, radiotherapy induces immunogenic cell death and causes cellular stress to increase the pool of tumor-associated antigens and damage-associated molecular patterns. Transactive dendritic cells are professional antigen-presenting cells acting with tumor-specific CD8+ T cells to enhance the anti-tumor responses and promote immune cell infiltration into the tumor microenvironment (21). Pro-tumoral and exhausted immune cells are observed in the immunosuppressive landscape of HCC and can convert an otherwise “cold” tumor with low immunogenicity and poor infiltration of immune cells to an immune-reactive “hot” one (22). Currently, many ongoing trials (NCT04167293, NCT03817736, NCT03203304, NC04611165, NCT03316872) are investigating whether radiotherapy combinations with immune checkpoint inhibitors will improve the survival of patients with HCC. For instance, a phase I trial (NCT02239900) combining liver or lung radiotherapy and ipilimumab demonstrated clinical benefit in 23% of patients. However, grade 3 toxicities were identified in 34% of participants, and no grade 4/5 toxicities were observed (23). Another phase I basket trial (NCT02608385) evaluated the safety of multi-site radiotherapy followed by pembrolizumab in patients with advanced solid tumors, including one case of HCC. The trial demonstrated this regimen’s potential efficacy and safety (24). More encouraging outcomes are expected from ongoing trials.

There are some limitations to our case report. A liver biopsy was not performed for pathologic diagnosis. Also, hepatitis B DNA quantification was not routinely monitored, neither were inflammatory markers, such as antigen-presenting cell or tumor-specific CD8+ T cells, assessed throughout the treatment process.

In conclusion, we report here a rare case of long-term survival of a patient with massive HCC treated with immunotherapy, anti-angiogenic therapy and radiotherapy. The patient was alive with more than 36 months lifetime after the episode, and longer dynamic follow-up will be conducted. This case confirms the feasibility of the combination of immunotherapy and anti-angiogenic therapy as neoadjuvant therapy, and indicates that immunotherapy with radiotherapy can act synergistically. More clinical trials are needed to confirm the non-invasive systematic treatment strategy, and more encouraging outcomes and the realization are expected.

## Data availability statement

The raw data supporting the conclusions of this article will be made available by the authors, without undue reservation.

## Ethics statement

The studies involving humans were approved by the Ethics Committees of Heyuan People’s Hospital. The studies were conducted in accordance with the local legislation and institutional requirements. Written informed consent for participation was not required from the participants or the participants’ legal guardians/next of kin in accordance with the national legislation and institutional requirements. Written informed consent was obtained from the individual(s) for the publication of any potentially identifiable images or data included in this article.

## Author contributions

YuL: Writing – original draft. YaL: Writing – original draft. XL: Writing – original draft. JW: Writing – original draft. KW: Writing – review & editing, Writing – original draft. HM: Writing – review & editing.
